# Differential proteomics of interstitial fluid in lung tissue associated with the progression of pulmonary fibrosis in mice

**DOI:** 10.1038/s41598-025-98569-w

**Published:** 2025-04-30

**Authors:** Xi Lu, Dong Han, Yifeng Nie, Yahong Shi, Tun Yan, Xiang Li

**Affiliations:** 1 College of Life Sciences, Bejing University of Chinese Medicine, Beijing, 100029 China; 2 CAS Center for Excellence in Nanoscience, National Center for Nanoscience and Technology, 100190 Beijing, P. R. China; 3 Institute of Medicinal Plant Development, Peking Union Medical College, Chinese Academy of Medical Sciences, 100193 Beijing, China; 4 College of Pharmacy, Baotou Medical College, 014040 Baotou, China

**Keywords:** Idiopathic pulmonary fibrosis, Fibrosis mechanism, Proteomics, Interstitial fluid, Computational biology and bioinformatics, Zoology, Biomarkers, Diseases

## Abstract

Idiopathic pulmonary fibrosis (IPF) is a chronic and fatal fibrosis disease. Due to the limited understanding of its pathogenesis and the fact that its detection largely depends on the operator’s technical level and the accuracy of the equipment, the diagnosis and treatment of the disease have significant limitations. In this research, bleomycin was used to establish IPF models of C57/BL6N mice with different injury degrees, and proteomics technology extracted interstitial fluid of lung tissue to analyze the mechanism of fibrosis at different stages. Compared with the normal group, the alveolar area, collagen deposition, tidal volume, and respiratory rate of the experimental group decreased at all periods, and the difference was most significant on the 14th day of modeling. Proteomic techniques, including gene ontology (GO) and Kyoto Encyclopedia of Genes and Genomes (KEGG) pathway enrichment, showed that the progression of pulmonary fibrosis was related to different pathways: glucose metabolism, lipid transport, glycoprotein metabolism, synthesis of sulfur compounds, and other energy metabolism, calcium ion transport were dominant in the early stage of fibrosis and the acute inflammatory stage. The endoplasmic reticulum stress pathway was dominant in the extreme stage of fibrosis, and blood flow shear stress, Extracellular matrix (ECM) receptor activation, and other extracellular matrix-related pathways were dominant in the late stage of fibrosis. Moreover, western bolt validation experiments also confirmed that C/EBP-homologous protein (CHOP), Heat Shock Protein 60 (HSP60), and Alpha smooth muscle actin(α-SMA) proteins were increased in expression related to this pathway at the extreme stage of fibrosis, suggesting that the disruption of ion balance in the endoplasmic reticulum induced by endoplasmic reticulum stress or the disturbance of protein processing and transportation were involved in the occurrence and development of pulmonary fibrosis in mice. The above results are expected to provide ideas for clinical interpretation of the mechanism of pulmonary fibrosis and provide vital data support for its accurate diagnosis and effective treatment.

**Supplementary Information:**

The online version contains supplementary material available at 10.1038/s41598-025-98569-w.

## Introduction

IPF is a chronic and fatal interstitial lung disease of unknown cause. It is characterized by progressive lung scar and histological images of interstitial pneumonia, dyspnea, and progressive deterioration of lung function, with a poor prognosis and a median survival of only 3–5 years from diagnosis^1–3^. IPF affects about 3 million people worldwide and has an incidence of 20–30 cases per 100,000 people and a prevalence of 10–60 cases per 100,000 people, with its incidence and prevalence increasing every year with age^4–8^. Clinically, patients with early pulmonary fibrosis may have no typical clinical manifestations or symptoms, only mild cough and dyspnea. The symptoms become gradually apparent with disease progression, including irritating dry cough, chest tightness, and asthma after exercise. When these symptoms occur, the patient’s lung interstitial disease is actually in a more serious state. Therefore, early detection and diagnosis are the key to improving the level of pulmonary fibrosis treatment. However, the pathogenesis of IPF is not yet clear, which greatly limits our comprehensive understanding and grasp of the disease’s nature and thus constitutes a significant obstacle to its accurate diagnosis and effective treatment.

**Fig. 1:**
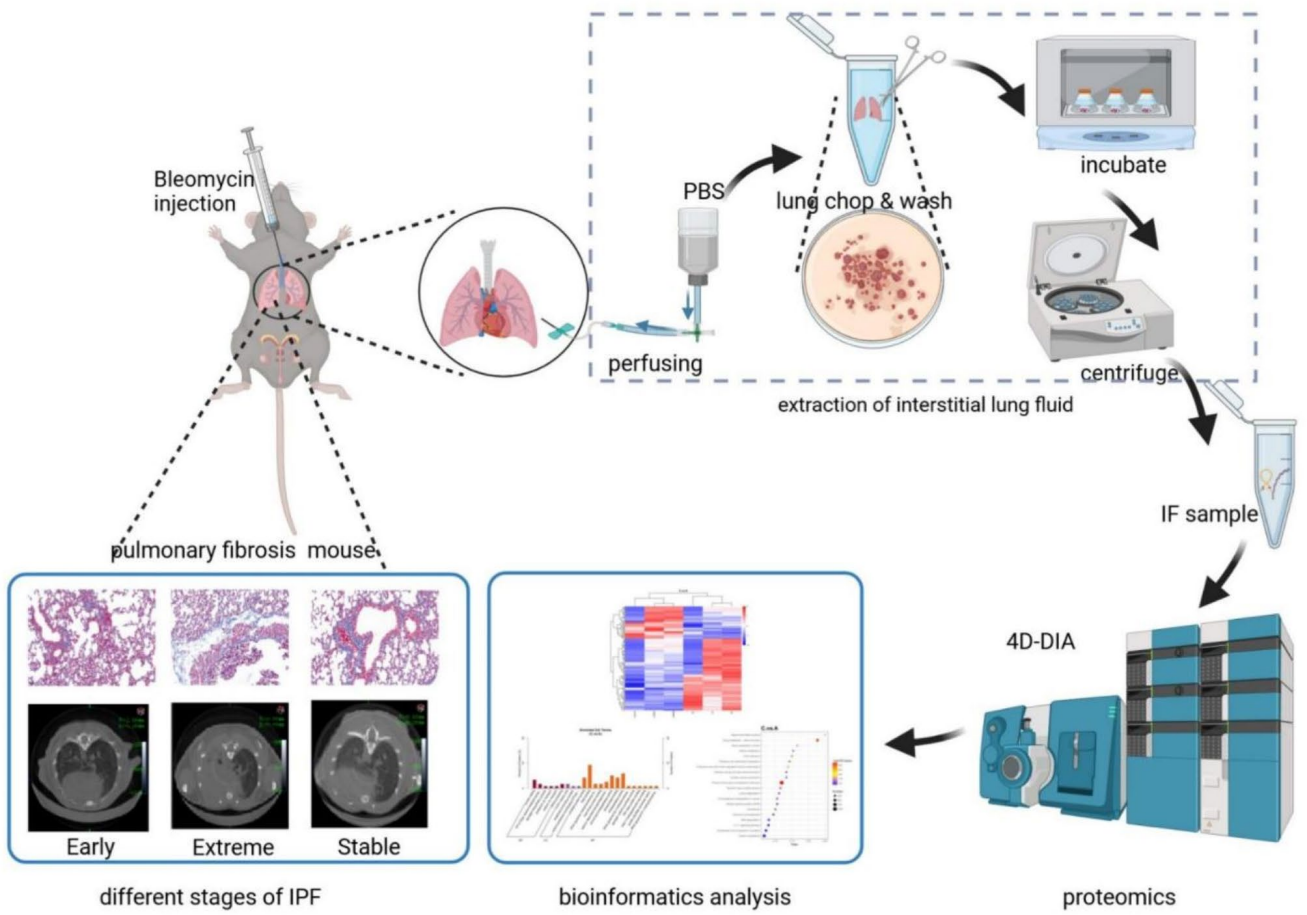
Construction of a pulmonary fibrosis mouse model and overall study flowchart of pulmonary interstitial fluid extraction and proteomics analysis.

The human body is composed of cells, tissues and other substantial structures wrapped by multi-level interstitium^9^. As the framework structure connecting the cells of the whole body, the interstitium structure is mainly composed of flowing matrix such as collagen fiber and hyaluronic acid. When the pulmonary interstitium structure undergoes fibrotic lesions, it will transform to the substantial lesions of cells and tissues. Currently, histological imaging of interstitial pneumonia and lung biopsy are the primary diagnostic methods of pulmonary fibrosis. The clinical diagnostic guidelines for IPF emphasize that its current diagnosis methods are highly dependent on high-resolution computed tomography (HRCT) volume scanning of the chest, which requires high accuracy of the instrument and highly skilled operators^2^. At the same time, lung biopsy has a risk of trauma. The study of pulmonary fibrosis biomarkers as a new way to diagnose pulmonary fibrosis that has the advantages of being noninvasive, sensitive, and accurate, is one of the hot areas in this field, including mucin-5 subtype B (MUC5B)^10^, matrix metalloproteinase-7 (MMP7)^11,12^, type II alveolar cell surface antigen (KL-6)^13^, chemokine ligand 18 (CCL18)^14^, much progress has been made. Interstitial fluid is a body fluid in the interstitial space that consists of proteins secreted by exosomes or via membrane protein shedding^15^. Compared to the whole-body circulating blood, the local interstitial fluids are closer to the lesion site where it is easier to capture the characteristic proteins of the disease. In addition, the high abundance of protein in interstitial fluid is less. Unlike blood, which is characterized by many interferences from proteins in other parts of the body, interstitial fluids have certain advantages in discovering potential biomarkers^16,17^. Therefore, the present study attempted to determine the injury effect of bleomycin-induced pulmonary fibrosis in mice at different time points. Hematoxylin-eosin (H&E) and Masson’s trichrome staining were used to observe cell morphology of lung tissue, calculate collagen deposition area, monitor tidal volume and respiratory rate, and evaluate pulmonary respiratory function. Changes associated with pulmonary fibrosis were determined using HRCT in animal lungs. Subsequently, the interstitial lung fluid of different groups were analyzed by data-independent acquisition (DIA) proteomics-based diaPASEF technology. Quantitative studies evaluated pulmonary interstitial fluid in mice with pulmonary fibrosis of different severity (Fig. 1). By studying the differential proteins and pathways related to the progression of pulmonary fibrosis through interstitial fluid proteomics, the mechanisms related to the occurrence and development of the disease are discovered. This can provide ideas and references for promoting the early diagnosis and treatment of pulmonary fibrosis and suggesting biomarkers related to its diagnosis.

## Methods and materials

### Construction of a pulmonary fibrosis mouse model

Healthy male C57BL/6 N mice, with an initial body weight of (18 ± 2) g, were obtained from Beijing Vital River Laboratory Animal Technology Co., Ltd. (License number: SCXK (Beijing) 2016-0011). The mice were housed in the SPF animal room at the National Nanoscience Center. The temperature in the animal room was maintained at (23 ± 2)℃, with humidity levels ranging from 40 to 60%. The animals had unrestricted access to food and water, and a 12-hour alternating light cycle simulated day and night conditions. Sixty male C57/BL6N mice aged five weeks were randomly divided into five groups, including the normal and modeling groups, and evaluated at 7, 14, 21, and 28 days. In the experiment, bleomycin solution diluted with phosphate-buffered saline (PBS) was injected into the trachea, with a final injection amount of 5 mg/kg body weight. The mice were thus induced to establish pulmonary fibrosis models via a single intratracheal injection. The main experimental materials and equipment were shown in the Supporting Information. This study is performed in accordance with relevant guidelines and regulations. All methods are reported in accordance with ARRIVE guidelines. The animal experiment scheme is reasonably designed, and the experimental techniques and purposes conform to human moral and ethical standards and international practices. Approval number: NCNST21-2106-0601.

### Lung function test

Different groups of mice were placed in the sample bin at different time points after the model was established. Once proper parameters were set after the mouse breathing was stable, the FinePointe Noninvasive detection system was used to determine the airway resistance (saw) of the awake experimental animals. The tidal volume and respiratory rate of the respiratory function were monitored.

### Micro-CT lung imaging

After modeling, mice in different groups at different time points were administered ventilation anaesthesia with 2.5% isoflurane and fixed on the operating table. Lung imaging was performed using the live animal imaging system (small animal high-resolution CT; model PE Quantum PE, USA). The images were analyzed with a three-dimensional finite element algorithm (AVATAR 1.5.0, PINGSENG, China).

### Histological staining

Six mice in each group were first overanesthetized with 2.5% pentobarbital sodium and then fixed with 4% paraformaldehyde administered in the left lung. After fixation with fixative solution and dehydration with gradient alcohol, the slices were permeabilized with xylene, embedded in paraffin, and sealed after staining. Finally, microscopic examination was performed and the images were collected and analyzed.

### Masson’s trichrome staining

First, samples were treated twice with xylene for 20 mins each time. Then, they were treated twice with 100% ethanol for 5 mins each time. Next, 95%, 90%, 80%, 75%, and 70% ethanol was used for 5 mins each time for gradient dehydration. The tissues were washed with distilled water for 5 mins and stained with hematoxylin. Finally, microscopically examined sections were collected and analyzed. In addition, mouse lungs were evaluated using ImageJ software and the area of lung collagen deposition was calculated to assess the degree of pulmonary fibrosis. For hematoxylin-stained nuclei, Weigert’s iron hematoxylin in the Masson staining kit should be treated for 5 mins, followed by running water. Hydrochloric acid alcohol differentiation treatment time is very short, only a few seconds, and then rinse with tap water and water for a few minutes to make it return to blue. Treat with ponceau acid fuchsin solution for 5–10 mins, then rinse quickly with distilled water; Then it is treated with an aqueous solution of phosphomolybdate for about 3–5 mins. Then aniline blue solution was used for re-dyeing, and the treatment was 5 mins. Then it was treated with 1% glacial acetic acid for 1 min; Then, they were treated with 95% ethanol twice for 5 mins each time and anhydrous ethanol for 5 mins each time. Finally, it was treated with xylene twice for 20 mins. After the treatment, it is dehydrated and permeated, and the slices are removed from the xylene to dry slightly and then sealed with neutral gum. Finally, microscopically examined sections were collected and analyzed. In addition, mouse lungs were analyzed using the Fiji distribution of ImageJ (version v2.3.0) software and the area of lung collagen deposition was calculated to assess the degree of pulmonary fibrosis.

### Extraction of interstitial lung fluid

In order to exclude the influence of high protein abundance in circulating blood on the results, lung tissue was perfused before sampling. In this study, fresh lung tissue was cut into 1–3 mm^3^ small pieces containing a protease inhibitor cocktail, placed in PBS containing protease inhibitors for careful cleaning, and then transferred to 15 mL tubules containing equal volume PBS. The samples were cultured in a cell incubator at 37℃ with 5%CO_2_ for 1 h. The samples were then taken and centrifuged 800 g for 5 mins, 2000 g for 10 mins, and 120,000 g for 30 mins. After the extracellular fluid was evenly distributed, gradient centrifugation was performed and the supernatant was filtered through a 200-mesh filter membrane to obtain pulmonary interstitial fluid. The interstitial fluid was then rapidly frozen in liquid nitrogen and placed in a -80 ℃ refrigerator until further proteomic examination.

### Proteomics and statistical analysis of lung interstitial fluid

The samples were transferred to a 1.5 mL centrifuge tube and an appropriate amount of denaturing buffer (DB) protein solution was added for shaking and mixing. After centrifugation, 10 mM dithiothreitol (DTT) was added to the supernatant and the reaction was performed at 56 °C for 1 h, followed by the addition of enough iodoacetamide (IAM). The reaction was performed at room temperature in the dark for 1 h. The extracted peptides were desalted and purified and the fractions were collected and analyzed by Bruker times of the Pro 2 high-resolution mass spectrometer after being incorporated into the internal retention time (iRT) standards. Some of the samples were first collected based on the data-dependent acquisition (DDA) model to establish the spectra library. The remaining samples were collected via the DIA model. After data collection, DDA database search and DIA analysis were performed by Spectronaut software(version 14.0, Biognosys)followed by statistical analysis. First, the protein difference analysis picks out the sample pairs that need to be compared and takes the ratio of the mean values of all biological replicates of each protein in the comparison sample pairs as the differential multiple (Fold Change, FC). To judge the significance of the difference, the relative quantitative values of each protein in the two comparison sample pairs are subjected to T-test, and the corresponding P-value is calculated, which is used as the significance index, and the default is *P-value* ≤ 0.05. GO annotation is to analyze the identified proteins by using the Interproscan software (version 5.56-89.0). This software involves the search of six well-known databases (Pfam, PRINTS, ProDom, SMART, ProCite, PANTHER) so that the annotation results will be more comprehensive. For instance, the GO functional significance enrichment analysis provides GO functional items with significantly enriched differential proteins compared to all identified protein backgrounds, thus indicating which biological functions the differential proteins are significantly associated with. Its specific derivation method:

$$\:P-value=\text{1}-\sum\:_{j=0}^{-1}\frac{\left(\frac{M}{j}\right)\left(\frac{N-M}{n-j}\right)}{\left(\frac{N}{n}\right)}$$

Among them, N is the number of proteins with GO annotation information among all proteins, n is the number of differential proteins in N, M is the number of proteins annotated to a GO entry among all proteins, and χ is the number of differential proteins annotated to a GO entry. Calculate the *P-value* value and use *P-value* ≤ 0.05 as the threshold. GO terms that meet this condition are defined as GO terms significantly enriched in differential proteins. The main biological functions of differential protein forms can be determined through GO significance analysis. The KEGG Pathway significance enrichment analysis method is the same as the GO functional enrichment analysis. The KEGG Pathway significance enrichment analysis method is similar to the GO functional enrichment analysis. Taking the KEGG Pathway^18^ as the unit, the hypergeometric test is applied to find the Pathways significantly enriched in the differential proteins compared with the background of all the identified proteins. The essential biochemical metabolic and signal transduction pathways involved in the differential proteins can be determined through the Pathway significance enrichment. GraphPad Prism 9.5 (version9.5.0, GraphPad Software, San Diego, CA, USA) was utilized for data visualization and further statistical analysis.

### Differential protein verification

The samples and marker were placed on ice, mixed by vortex shaking after dissolution, and centrifuged for 10 s at 4,000 rpm. The glass plate was fixed in the electrophoresis tank and the electrophoresis liquid was added to the electrophoresis tank to the specified height. The sample size was calculated based on the sample protein concentration, and the corresponding sample and marker were added to the sampling tank according to the experimental design for electrophoresis. The membrane was transferred using the transfer liquid and the protein bands were observed using western blot imager. The protein expression levels were analyzed according to the grey values of the bands. Adobe Illustrator 2022 (version v26.3.1.1103, Adobe Systems Incorporated, San Jose, CA, USA) was used for the final preparation of figures for publication.

### Statistical analysis

For statistical analysis, we used IBM SPSS Statistics for Windows (version 27.0, IBM Corp., Armonk, NY, USA). All data were expressed as mean ± standard deviation. One-way analysis of variance (ANOVA) was used to evaluate the statistical significance of the differences among the groups. Least—Significant Difference (LSD) correction was used to make multiple comparisons between the groups, where *P* < 0.05 was considered statistically significant.

## Results

### Construction of a pulmonary fibrosis mouse model and measurement of the fibrosis damage effect

**Fig. 2:**
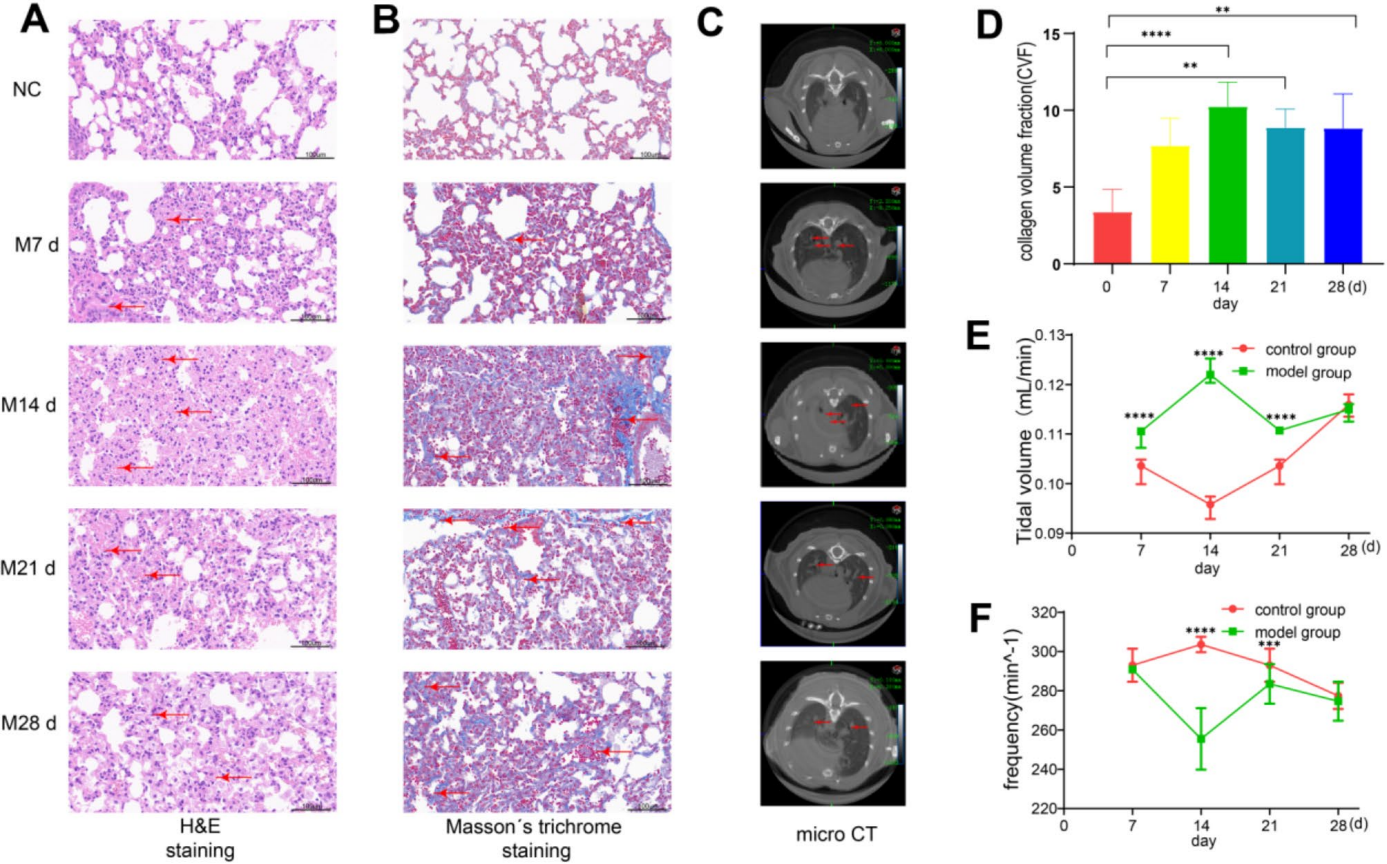
Effect of bleomycin on pulmonary fibrosis injury in mice. (**A**) H&E staining images of lung tissue in each group of mice. (**B**) Masson’s staining images of lung tissue in each group of mice. (**C**) CT imaging characteristics of lungs in each group of mice: (**D**) ratio of collagen deposition area in lung tissue; (**E**) respiratory tidal volume; and (**F**) respiratory frequency(*n* = 6). *P<0.05, **P<0.01, ***P<0.001, ****P<0.0001.

The pulmonary fibrosis model of C57/BL6N mice induced by bleomycin was thus established. HRCT and lung function evaluation were performed in mouse lungs before sampling at different time points (7, 14, 21, and 28 days of modeling). Six mice in each group were randomly selected for H&E and Masson’s trichrome staining (Fig. 2A and B). Compared to the normal group, the alveolar area of mice in the modeling group was reduced and the inflammatory infiltration and pulmonary interstitial fiber deposition were increased. The lung HRCT results showed that the lungs of mice with pulmonary fibrosis showed typical cable-like and grid-like high-density shadows, where the damage was most serious on day 14 of modeling (Fig. 2C). Collagen deposition area ratio was also calculated (Fig. 2D). The pulmonary function test results showed that compared to the normal group, The tidal volume was decreased (Fig. 2E). the respiratory rate of mice in the model group was accelerated, and this difference was most apparent on the 14th day of modeling (Fig. 2F). Lungs were extracted from the remaining mice followed by extraction of pulmonary interstitial fluid for subsequent experiments.

### Detection and proteomic analysis of different pulmonary fibrosis mice

**Fig. 3:**
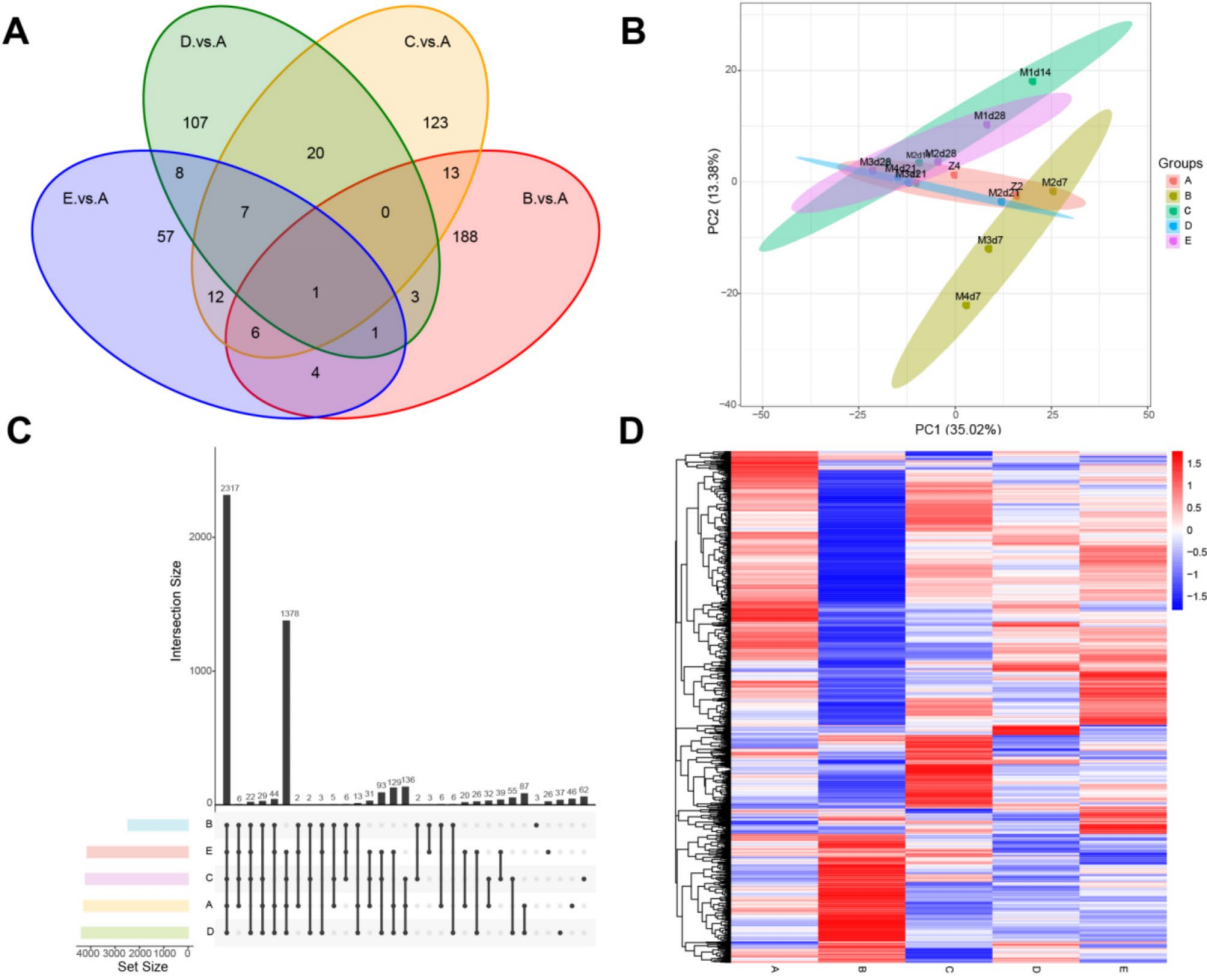
Proteomic analysis of the effect of bleomycin on pulmonary fibrosis injury in mice. Protein statistical analysis of pulmonary interstitial fluids in the normal, 7-, 14-, 21-, and 28-day modeling groups. The sample proteins were displayed using (**A**) differential protein Wayne plots, (**B**) principal component analysis (PCA), (**C**) UpSetR plots, and (**D**) clustering heatmap. The Wayne plot represents differential proteins between different groups, while PCA reflects the overall protein differences between each group of samples and the variability between samples within the group. The clustering heat map represents the up-regulation and down-regulation of different proteins compared between different products(*n* = 3). (Grouping: (**A**) normal group, (**B**) 7-day modeling group, (**C**) 14-day modeling group, (**D**) 21-day modeling group, and (**E**) 28-day modeling group).

The lungs of different mice with pulmonary fibrosis were extracted and interstitial fluid of lung tissue was extracted. DIA technology was used for proteomic analysis to screen out differential proteins that could be used as biomarkers and explain their potential mechanisms. The mechanism and biological markers of the difference of lung interstitial fluid between normal group and fibrotic model group were studied by proteomics. The Spectronaut pulsar module searches and analyzes spectral data from the Spectronaut Raw file. Import spectral data into Spectronaut to build a real spectrum and DDA library. The protein was identified after comparison. The Wayne diagram of differential proteins shows the different proteins in different groups. The results show that the common differential protein in each group is Mettl21A, which is related to endoplasmic reticulum stress (Fig. 3A). Principal component analysis (PCA) analysis showed that there was significant separation of differential proteins between the normal group and the pulmonary fibrosis injury group (Fig. 3B). UpSetR plots showed the common and specific partial differences between groups (Fig. 3C). The results showed that 4666 proteins were detected in lung interstitial fluid proteomics (Fig. 3D). The subcellular localization of the different proteins in the five groups was examined (Fig. S1). Five groups’ total proteins were subjected to enrichment analyses, including GO, IPR of total protein, KEGG, and COG. (Fig. S2-S5). A Venn diagram was created to show the protein enrichment in the five groups (Fig. S6).

### Enrichment annotation analysis of KEGG and GO pathways

In studying pulmonary fibrosis in mouse models, analyzing differentially expressed proteins (DEPs) at various post-modeling points provides crucial insights into the disease’s progression. Comparing the interstitial fluid proteomes of pulmonary fibrosis mice with those of the normal group at 7, 14, 21, and 28 days after modeling can comprehensively understand the underlying biological mechanisms.

**Fig. 4:**
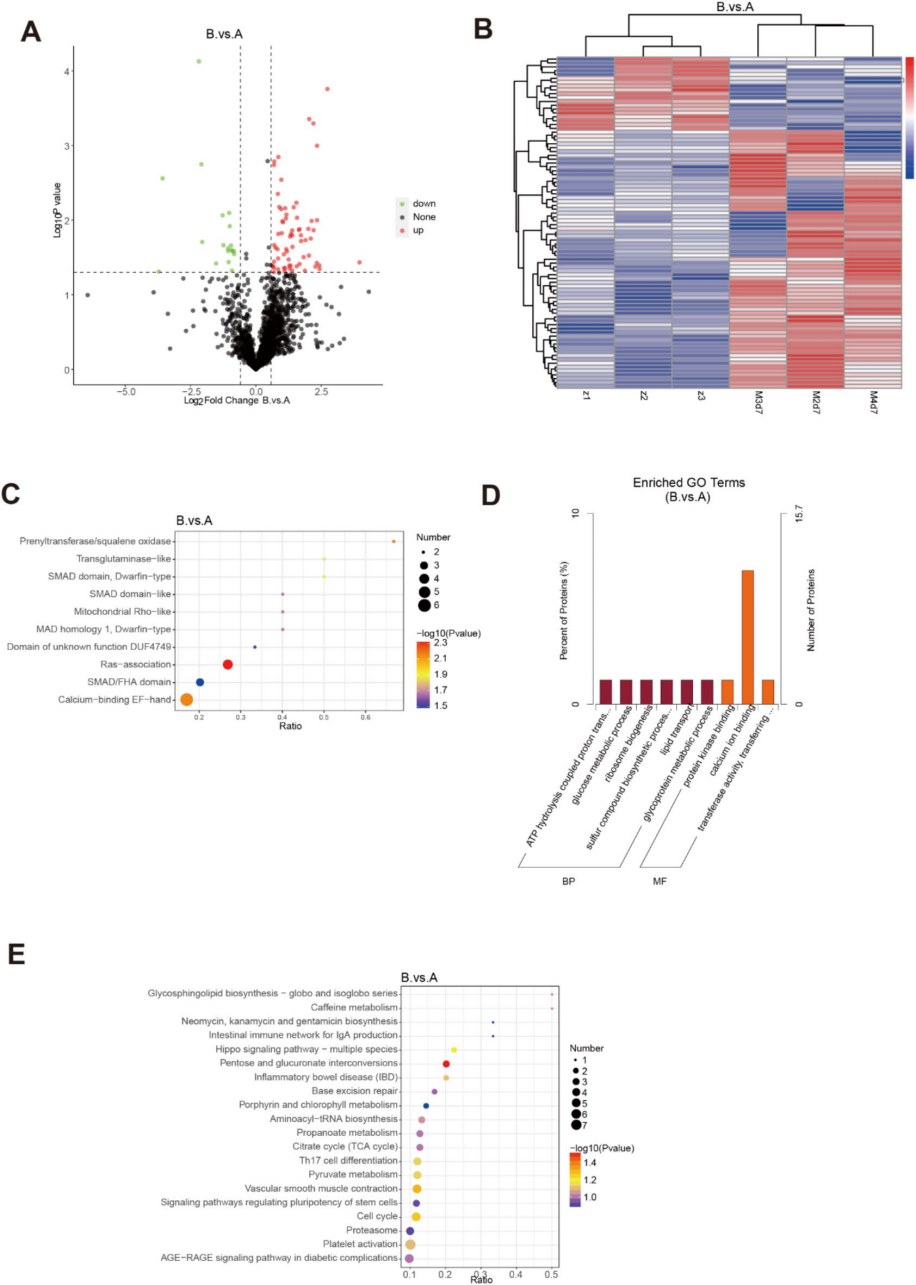
Comparison of differential genes and enrichment pathways between normal control group and 7-day model group. The differential proteins in the sample were shown with (**A**) volcano maps and (**B**) heat maps. GO, KEGG and IPR enrichment explain the underlying mechanism of early onset of pulmonary fibrosis. (**C**) The difference protein IPR enrichment analysis between the normal group and the model group showed that it was related to the heat shock protein family. (**D**) Differential protein GO enrichment analysis between normal control group and modeling group. (**E**) KEGG enrichment analysis between normal control group and model group was mainly concentrated in endoplasmic reticulum stress pathway (*n* = 3). (Group: A normal group, C 7-day model group).

At 7 days after modeling, 216 DEPs were identified in the interstitial fluid of pulmonary fibrosis mice. Among them, 69 proteins were up-regulated, and 147 were down-regulated (Fig. 4A and B). Database-based functional annotation revealed that energy metabolism and calcium channel-related pathways were the most significantly enriched (Fig. 4C-E)^19–23^. This finding indicates that energy production and calcium ion regulation processes play essential roles in the early stages of pulmonary fibrosis, which are likely associated with the initial inflammation and fibrosis development. Additionally, InterPro Resource-enriched (IPR-enriched) and GO-enriched differential proteins were involved in biological processes such as calcium ion channels, acyltransferase activation, protein kinase binding, glucose metabolism, lipid transport, glycoprotein metabolism, and sulfur-containing compound synthesis. KEGG enrichment analysis showed significant differences in pathways like vascular smooth muscle contraction, cell cycle, HIPPO signaling pathway, pyruvate metabolism, and cell differentiation between the normal and model groups.

**Fig. 5:**
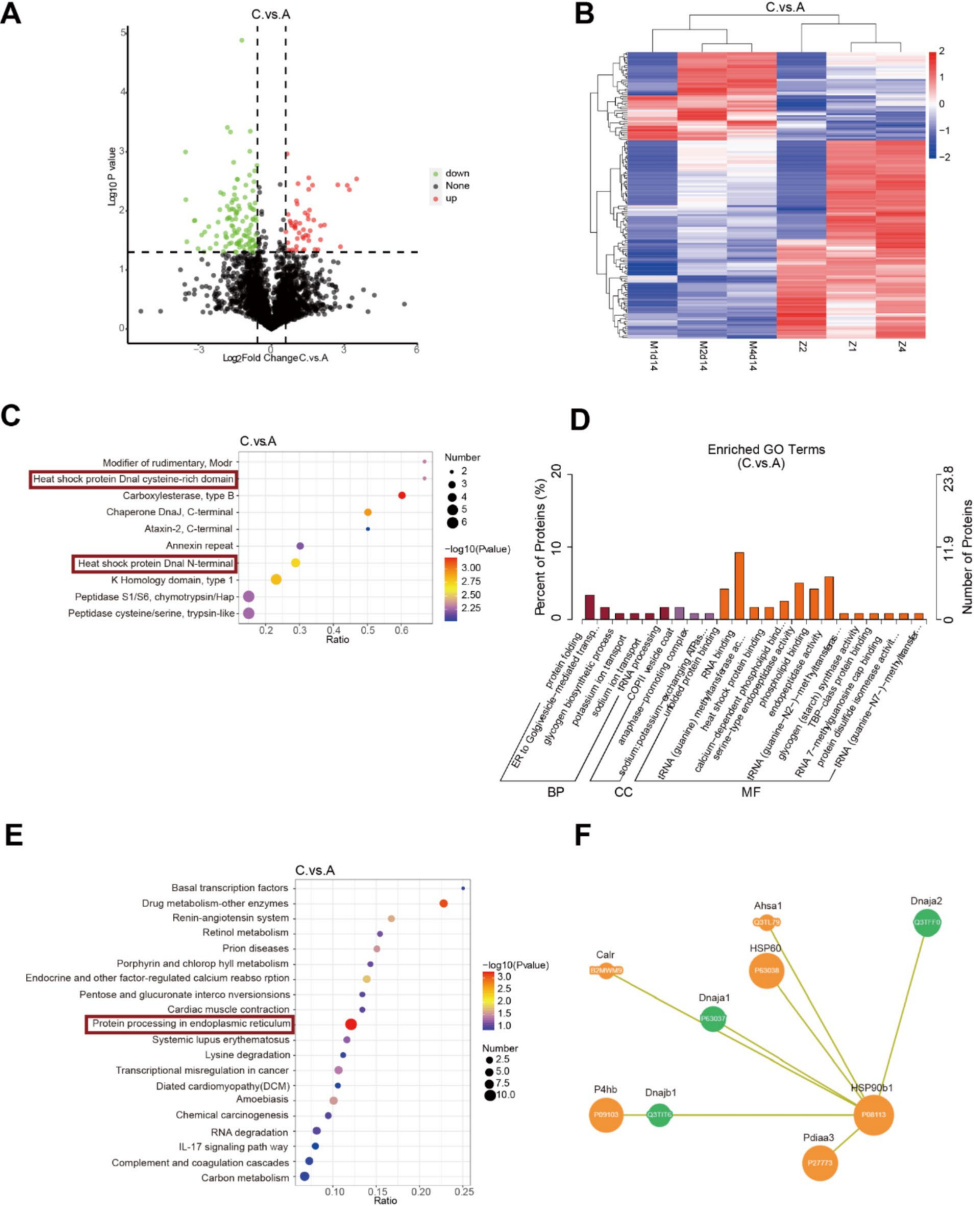
Comparison of differential genes and enrichment pathways between the normal control and 14-day modeling group. The differential proteins in the sample were displayed using (**A**) volcano map and (**B**) heatmap. GO, KEGG, and IPR enrichment explain the potential mechanisms of pulmonary fibrosis occurrence. (**C**) Enrichment analysis of differential protein IPR between the normal and modeling groups showed a correlation with the heat shock protein family. (**D**) Enrichment analysis of differential protein GO between the normal control and modeling groups. (**E**) KEGG enrichment analysis of the differential proteins between the normal control and modeling groups was mainly enriched in the endoplasmic reticulum stress pathway. (**F**) Correlation between heat shock proteins in the endoplasmic reticulum stress pathway(*n* = 3). (Grouping: (**A**) normal and (**C**) 14-day modeling groups).

After 14 days after modeling, 182 DEPs were detected, with 59 up-regulated and 123 down-regulated proteins (Fig. 5A and B). IPR enrichment analysis showed a significant expression of heat shock proteins (Fig. 5C). GO enrichment indicated that the DEPs were mainly involved in protein folding, mediating endoplasmic reticulum transport to Golgi vesicles, and glycogen biosynthesis (Fig. 5D). KEGG enrichment analysis revealed that the differences between the normal and model groups were mainly concentrated in the endoplasmic reticulum stress, drug metabolism, and signal transduction pathways mediated by calcium-sensitive receptors (Fig. 5E). The GO enrichment analysis further emphasized the enrichment of the endoplasmic reticulum stress pathway, and the protein-protein interaction map demonstrated the relationship between heat shock proteins and endoplasmic reticulum stress-related proteins. The protein-protein interactions of differential proteins between the normal and model groups were analyzed 14 days after modeling (Fig. S7). This suggests that at this stage, the endoplasmic reticulum stress response and protein-folding-related processes are crucial in the progression of pulmonary fibrosis (Fig. 5F).

**Fig. 6:**
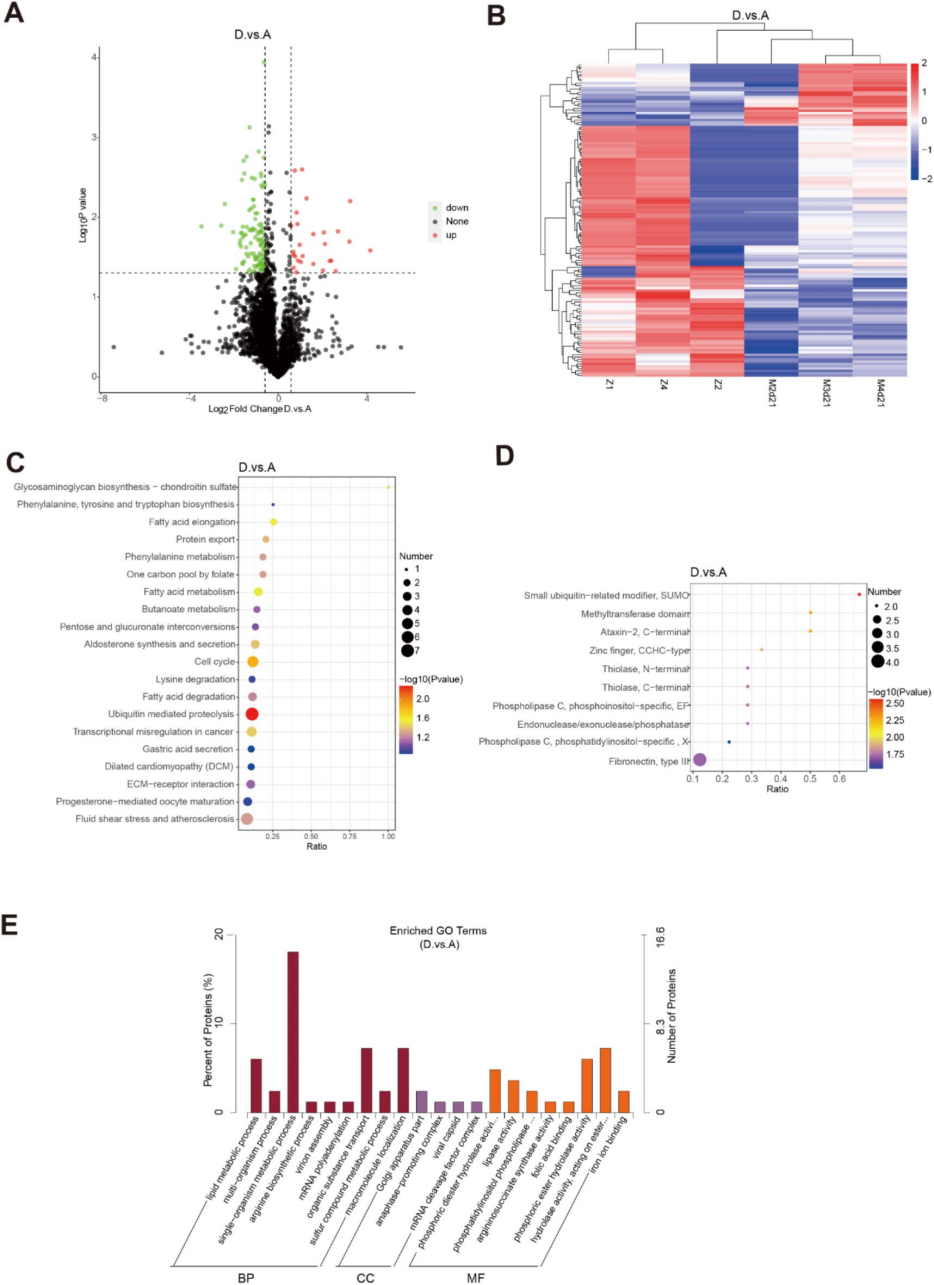
Comparison of differential genes and enrichment pathways between normal control group and 21-day model group. The differential proteins in the sample were shown with (**A**) volcano maps and (**B**) heat maps. GO, KEGG, and IPR enrichment explain the underlying mechanisms of matrix remodeling during the recovery phase of pulmonary fibrosis. (**C**) KEGG enrichment analysis between normal control group and model group was mainly concentrated in endoplasmic reticulum stress pathway. (**D**) The difference protein IPR enrichment analysis between the normal group and the model group showed that it was related to the heat shock protein family. (**E**) Differential protein GO enrichment analysis between normal control group and modeling group (*n* = 3). (Group: A normal group, D 21-day model group).

At 21 days after modeling, 147 DEPs were identified, with 36 up-regulated and 111 down-regulated proteins (Fig. 6A and B). The database was used to label the functions of the above identified proteins and understand the functional characteristics of different proteins (Fig. 6C-E). KEGG enrichment analysis showed that the differences between the normal and model groups were mainly reflected in pathways such as ubiquitin-mediated proteolysis, cell cycle, fatty acid metabolism, glycogen oligosaccharide synthesis, extracellular matrix (ECM) - receptor interaction, fluid shear stress, and atherosclerosis. IPR enrichment differences were mainly in fibronectin, small ubiquitin, and methyltransferase. GO-enriched proteins were significantly expressed in lipid metabolism, virion synthesis, single-biological metabolism, and arginine biosynthesis. These results imply that at this stage, processes related to protein degradation, cell-matrix interactions, and lipid metabolism contribute to the development of pulmonary fibrosis.

**Fig. 7:**
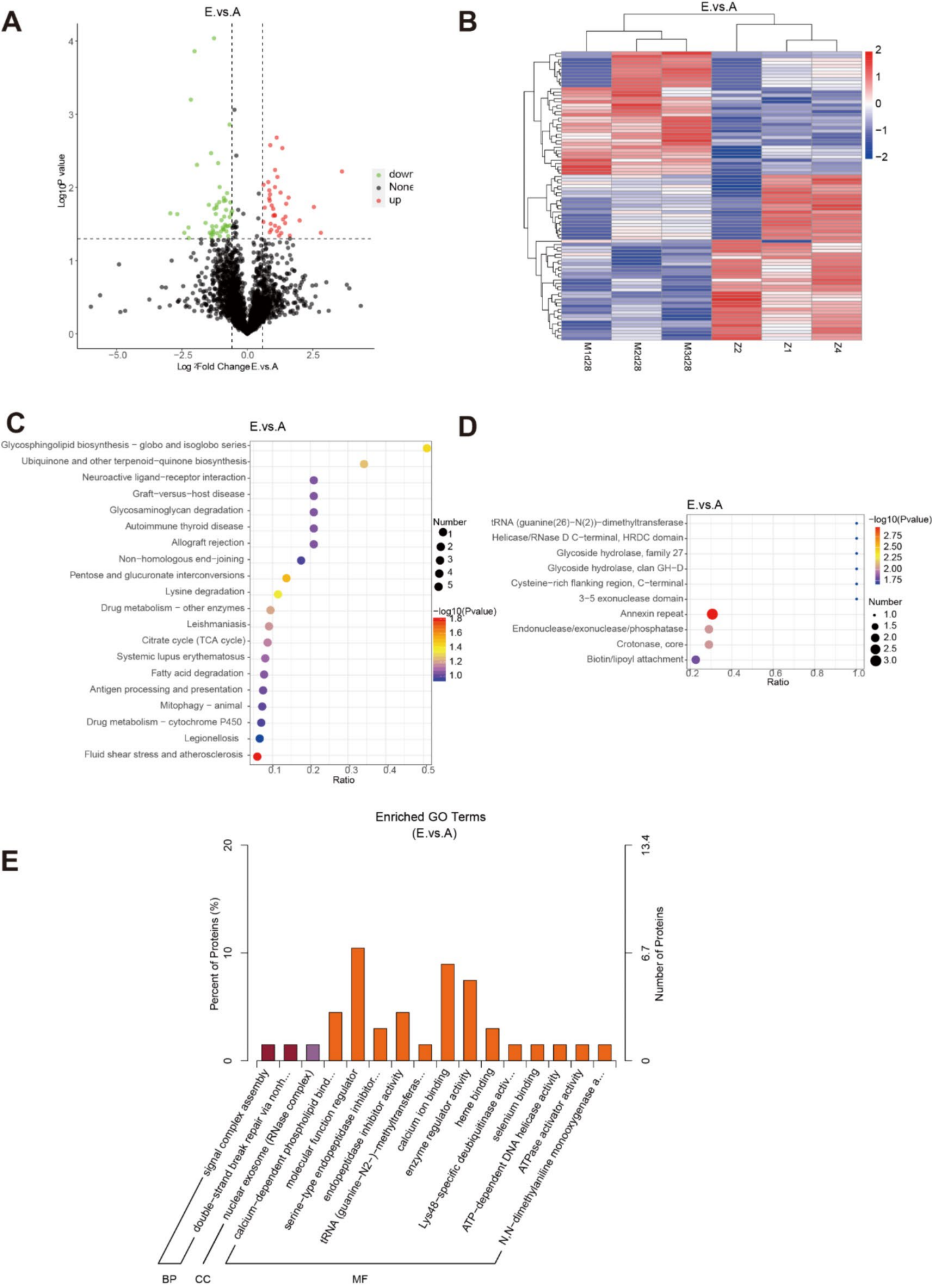
Comparison of differential genes and enrichment pathways between normal control group and 28-day model group. The differential proteins in the sample were shown with (**A**) volcano maps and (**B**) heat maps. GO, KEGG and IPR enrichment explain the underlying mechanism of pulmonary fibrosis. (**C**) KEGG enrichment analysis between normal control group and model group was mainly concentrated in endoplasmic reticulum stress pathway. (**D**) The difference protein IPR enrichment analysis between the normal group and the model group showed that it was related to the heat shock protein family. (**E**) Differential protein GO enrichment analysis between normal control group and modeling group (*n* = 3). (Group: A normal group, E 28-day model group).

At 28 days after modeling, 96 DEPs were found, with 43 up-regulated and 53 down-regulated proteins (Fig. 7A and B). KEGG enrichment analysis showed that the differences were mainly in pathways like fluid shear stress and atherosclerosis, pentose and glucuronic acid conversion, sphygmatolipid biosynthesis, lysine degradation, ubiquinone and other terpenoid quinones biosynthesis, drug metabolism-other enzymes, and the citric acid cycle (Fig. 7C). IPR enrichment differences were mainly in Annexin, endonuclease, and crotonase (Fig. 7D). GO-enriched proteins were significantly expressed in calcium-dependent phospholipid binding and calcium-ion binding (Fig. 7E). This suggests that at the later stage of pulmonary fibrosis, processes related to lipid and carbohydrate metabolism, as well as calcium-related functions, are essential in the disease’s progression.

In summary, as pulmonary fibrosis progresses in the mouse model, the functional characteristics of DEPs change over time. Early stages are dominated by energy metabolism and calcium-related processes, while later stages involve more complex pathways related to protein degradation, metabolism, and cell-matrix interactions. These findings contribute to a better understanding of the molecular mechanisms underlying pulmonary fibrosis and may provide potential therapeutic targets for the disease.

### Verification and mechanism of differential proteins in pulmonary interstitial fluid of mice with pulmonary fibrosis

**Fig. 8:**
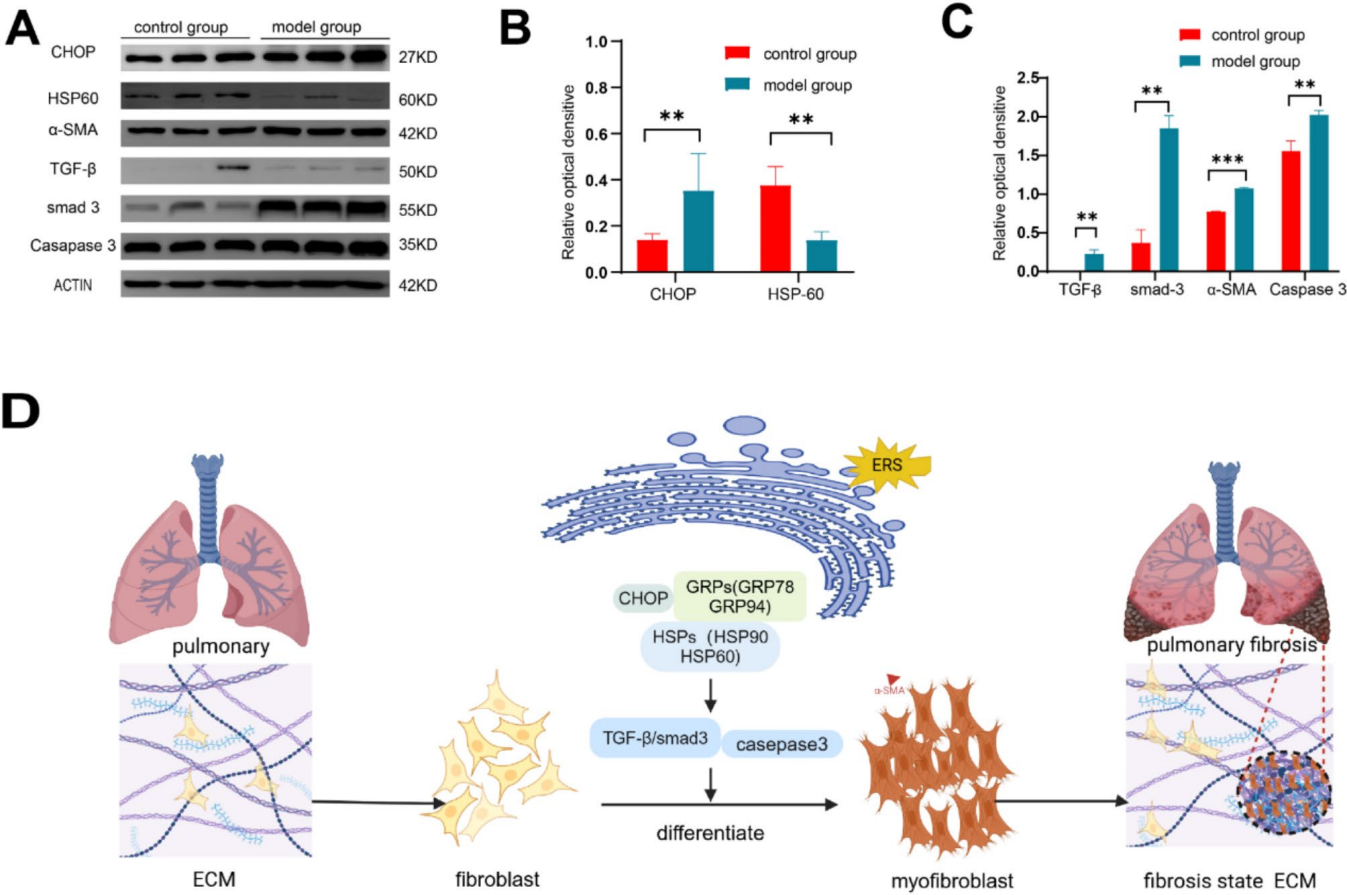
(**A**) Western blot validation of differentially expressed proteins in the interstitial fluid of pulmonary fibrosis mice. (**B**,**C**) Expression levels of differentially expressed proteins in the normal control and 14-day modeling group mice. (**D**) Schematic diagram of the mechanism of endoplasmic reticulum stress-induced lung fiber injury in mice(*n* = 3). *P<0.05, **P<0.01, ***P<0.001, ****P<0.0001.

When bleomycin was injected into mice to establish a pulmonary fibrosis model, the endoplasmic reticulum-related pathways were activated and endoplasmic reticulum stress led to changes in protein synthesis and folding in cells, which affected alveolar epithelial cells, lung macrophages, and fibroblasts. Biological experiments showed that compared to the normal group, CHOP, transforming growth factor-β1 (TGF-β1), Smad3, alpha smooth muscle actin (α-SMA), and caspase-3 proteins were up-regulated, while HSP-60 protein was down-regulated in the modeling group (Fig. 8A-C and S8). Proteomic analysis revealed that endoplasmic reticulum stress-induced fibroblast differentiation into myofibroblasts increased α-SMA expression and collagen synthesis. At the same time, caspase3, CHOP and other apoptotic pathways were activated, resulting in apoptosis of alveolar epithelial cells (Fig. 8D). CHOP can also induce pulmonary macrophage M2-type polarization in bleomycin-induced pulmonary fibrosis model mice, while M2-type macrophages can promote pulmonary fibrosis by secreting pro-fibrotic factors, such as TGF-β, chemokines, and MMPs^24^ .

## Discussion

Idiopathic pulmonary fibrosis (IPF) is difficult to reverse due to its unknown pathogenesis and rapid disease progression. The five-year survival rate of patients after diagnosis is less than 30%. Therefore, early detection and diagnosis are crucial for improving the cure level of pulmonary fibrosis. At present, interstitial fluid proteomic analysis shows that it has a unique advantage compared to biomarkers found in serum^16,17^. For example, through in-depth proteomic analysis of liver cancer parenteral fluid, Jian Zhang’s team discovered a novel liver cancer candidate serum marker that is more sensitive and accurate than alpha-fetoprotein and represents the successful application of interstitial fluid in the discovery of cancer serum biomarkers^16^.In this study, a mouse model of pulmonary fibrosis was established by intratracheal injection of bleomycin. Combined with detection methods such as pathological sections, lung function, and lung CT imaging, it reflects the pathological characteristics of different stages and periods in the process of pulmonary fibrosis.

In the acute inflammation and early fibrosis stage, seven days after modeling, energy metabolism pathways such as glucose metabolism, lipid transport, glycoprotein metabolism, sulfur-containing compound synthesis, and calcium ion channels are the most enriched compared with the normal group. This is related to the early inflammation and fibrosis of pulmonary fibrosis. On the one hand, lung diseases such as pulmonary fibrosis may destroy lung tissue structure and cause a decline in cell function, affecting normal energy metabolism. On the other hand, abnormal energy metabolism may also promote the occurrence and development of diseases. For example, abnormal glucose metabolism is closely related to the occurrence of pulmonary fibrosis^19^. The intermediate products produced during glucose metabolism can stimulate lung tissue and aggravate the inflammatory response, leading to pulmonary fibrosis. In addition, glutamine decomposition also plays a vital role in pulmonary fibrosis^19^. Inhibiting glutaminase (GLS1) can reduce bleomycin-induced pulmonary fibrosis, indicating that regulating the glutamine metabolism pathway may have therapeutic potential for pulmonary fibrosis^25^. The metabolites of glutamine decomposition, such as α-ketoglutaric acid (α-KG), may also participate in the pathological process of pulmonary fibrosis. A continuous inflammatory response accompanies the process of pulmonary fibrosis and requires energy support. At the same time, signal pathways related to calcium ions, such as calcium ion transport, have also been found. In recent years, researchers have also found that mitochondrial calcium single receptors (MCUS) regulate the metabolic reprogramming of lung macrophages and the process of pulmonary fibrosis^26^. Therefore, the energy metabolism disorder may aggravate the inflammatory response in the lungs and further promote the progression of pulmonary fibrosis.

In the extreme stage of pulmonary fibrosis injury, on the 14th day after modeling, there are 169 different proteins in the interstitial fluid of mice with pulmonary fibrosis compared with the normal group, including 120 up-regulated proteins and 49 down-regulated proteins. The GO enrichment results of proteomics show that the two groups’ biological processes of different protein expressions are significantly different, including protein folding, endoplasmic reticulum transport to Golgi vesicles, and glycogen biosynthesis. The differences in KEGG enrichment mainly manifest in endoplasmic reticulum stress, drug metabolism, and calcium-sensing receptor-mediated signal transduction pathways, among which the endoplasmic reticulum stress pathway is the most significant. Subsequent biological experiments also showed increased expression of endoplasmic reticulum stress pathway-related proteins such as CHOP and HSP60 and fibroblast activation-related proteins such as α-SMA. These results indicate that the formation mechanism of IPF may involve endoplasmic reticulum stress-mediated differentiation of fibroblasts into myofibroblasts, synthesis of abundant collagen fibers, and accumulation in the extracellular matrix (ECM). It has been found that the endoplasmic reticulum is the protein processing workshop of the body cells, and its main functions include protein synthesis, folding, assembly and transportation, as well as the degradation of defective proteins^27–29^. Protein folding in the endoplasmic reticulum is a highly complex process influenced by a multitude of factors, and interference by any factor can lead to endoplasmic reticulum stress (ERS)^30,31^. In recent years, it has been found that endoplasmic reticulum stress is related to the occurrence and development of fibrotic diseases including IPF^32^. When fibrosis occurs, the profibrotic effect of endoplasmic reticulum stress can be conducted by many different types of cells in the lung, including fibroblasts^32,33^, alveolar epithelial cells, and macrophages, and finally recruit fibroblasts and promote their differentiation into myofibroblasts^34,35^, thereby activating the TGF-β/Smad signaling pathway and promoting fibrosis^36,37^. Pathological mesenchymal cells release a large amount of ECM, eventually leading to pulmonary fibrosis^32,38,39^. Expressions of heavy-chain binding protein (Bip), Endoplasmic Reticulum degradation-enhancing α-mannosidase-like protein (EDEM), X-box binding protein 1 (XBP1), Activating transcription factor 4 (ATF4), Activating transcription factor 6 (ATF6) and CHOP were increased in Alveolar Epithelial Cells (AEC) of IPF patients, accompanied by Caspase-3-induced apoptosis^39,40^. In addition, endoplasmic reticulum stress affects the function of alveolar epithelial cells, decreasing repair ability and promoting pulmonary fibrosis. This study found that key proteins related to endoplasmic reticulum stress are involved in the pathogenesis of pulmonary fibrosis. ER-, JNK- and CHOP-mediated apoptosis pathways can activate stress, promote inflammation, release TGF-β1 to promote fibrosis, activate α-SMA, and promote fibroblast to myofibroblast transformation. Protein verification results showed that CHOP, TGF-β1, Smad3, α-SMA and apoptosis-related caspase-3 proteins were up-regulated in the model group compared with the normal group, suggesting that the pathogenesis of bleomycin-induced pulmonary fibrosis in mice is related to the regulation of endoplasmic reticulum stress pathways.

In the stable stage of pulmonary fibrosis injury, on the 21st and 28th days after modeling, there are significant differences in interstitial fluid proteomics between the two groups in pathways related to pentose and glucuronic acid conversion, lysine degradation, fluid shear stress, ECM receptor interaction, and other cellular mechanical microenvironment. These are related to tissue repair and spatial reorganization of collagen fibers during the recovery period of pulmonary fibrosis injury. The interaction between ECM receptors and blood flow shear stress in the fibrosis process is a complex and crucial biomechanical and biological phenomenon. Understanding how cells perceive and transmit the evolution of mechanical transduction pathways interacting with extracellular matrix and hemodynamics is a common biomechanical feature of multi-organ fibrosis^41^. ECM plays a vital role in generating and transmitting mechanical signals to cells^42,43^. The characteristics of fibrotic tissue are increased elasticity, enhanced ECM cross-linking^44^, and reduced viscosity^17,45^. Excessive deposition and formation of fibers are superior to the dissolution of fibers, preventing the degradation of excessive matrix^46^ and providing a material basis for the mechanical stability of ECM. In addition, ECM cross-linking regulates tissue viscoelasticity by advanced glycation end products (AGEs), transglutaminase (TGase), and lysine oxidase (LOX)^47^. The vicious circle between fibrosis expansion, hemodynamic stress, and microvascular remodeling leads to irreversible tissue fibrosis. Appropriate shear stress can maintain endothelial homeostasis. Low Shear Stress (LSS) in the fibrotic microvascular system promotes endothelial dysfunction, leading to increased fibroblast activation, cadherin, vimentin, Anti - mitochondrial Antibodies (AMA) secretion, and collagen deposition^48^. In fibrotic diseases (such as liver fibrosis, pulmonary fibrosis, etc.), the interaction between ECM receptors and blood flow shear stress may be one of the essential driving forces for disease progression.

## Conclusion

This study established a mouse model of pulmonary fibrosis by bleomycin and combined multiple detection methods, providing valuable insights into the disease progression and potential treatment strategies of pulmonary fibrosis. Although the mouse model may not wholly replicate human pulmonary fibrosis, it still has essential significance. The interstitial fluid of lung tissue suggests different mechanisms at different stages of the occurrence and development of mouse pulmonary fibrosis, and various treatment plans can be formulated according to different injury stages. In addition to conventional cytokine pathway treatment, attention should be paid to regulating mechanical transduction, perception, and mechanical stimulation on cell behavior during the fibrosis process and their influence on the pulmonary fibrosis process through mechanical crosstalk. Combined with protein verification results, it is found that pulmonary fibrosis may be related to endoplasmic reticulum stress and the heat shock protein family. In addition, the heat shock protein (HSP) family includes heat emergency protein types that are widely present in mammals, and the HSP90 protein has been used as a key biomarker for various diseases, including tumors and neurological disorders^49,50^. Among them, HSP60, as a heat shock family protein associated with the endoplasmic reticulum stress pathway, is closely related to mouse pulmonary fibrosis and has the potential as a biomarker for its detection and diagnosis. Relevant follow-up clinical verification work is currently underway. This study will provide an appropriate reference and idea for the accurate diagnosis and effective treatment of clinical pulmonary fibrosis.

## Electronic supplementary material

Below is the link to the electronic supplementary material.

Supplementary Material 1

## Data Availability

The datasets generated during the current study are available from the corresponding author upon reasonable request.
