# Supplementary material for: Differential proteomics of interstitial fluid in lung tissue associated with the progression of pulmonary fibrosis in mice
Source: Sci Rep. 2025 Apr 30;15:15255. doi: 10.1038/s41598-025-98569-w (PMC12043939; doi:10.1038/s41598-025-98569-w)
Supplement: Supplementary file 1 — Supplementary Material 1 [file 41598_2025_98569_MOESM1_ESM.docx]

**Differential proteomics of interstitial fluid in lung tissue associated with the progression of pulmonary fibrosis in mice**

*Xi Lu^1,2,+^, Dong Han^1,2,+^, Yifeng Nie^2,^*, Yahong Shi^3^, Tun Yan^4^, Xiang Li ^2,^**

^1^College of Life Sciences, Bejing University of Chinese Medicine, Beijing 100029, China

^2^CAS Center for Excellence in Nanoscience, National Center for Nanoscience and Technology, Beijing, 100190, P.R China.

^3^Institute of Medicinal Plant Development, Peking Union Medical College and Chinese Academy of Medical Sciences, Beijing 100193, China.

^4^College of Pharmacy, Baotou Medical College, Baotou 10040, China.

^+^These authors contributed equally to this work.

*Corresponding Author: Yifeng Nie and Xiang Li.

CAS Center for Excellence in Nanoscience, National Center for Nanoscience and Technology, Beijing, 100190, P.R China.

1. mail address: nieyf@nanoctr.cn, lixiang@nanoctr.cn.

1.1 Required drugs and reagents

Bleomycin /Zeocin (50mg/ml) is purchased from Guona Chenyu (Beijing) Technology Co., LTD., brand Biofeng Lab. Isoflurane (100ML/ bottle) purchased from Rayward Life Technology (Shenzhen) Co., LTD., brand Rayward RWD. PBS, pentobarbital sodium, 4% paraformaldehyde, iRT kit (purchased from Biognosys), Bradford Protein Quantifying Kit (purchased from Biyantian), dithiothreitol (DTT, purchased from Sigma/D9163-25G), iodoacetamide (IAM, Purchased from Sigma/I6125-25G), Sodium dodecyl sulfate (SDS, Purchased from Sinopyma), urea (purchased from Sinopyma /10023218), mass spectrum grade pancreatic enzyme (purchased from Promega/V5280), ammonium bicarbonate (purchased from Sigma/5330050050), LC-MS grade ultra-pure water (purchased from Thermo Fisher) Chemical/W6-4), triethylammonium bicarbonate buffer (TEAB, Purchased from Sigma/T7408-500ML), LC-MS grade acetonitrile (purchased from Thermo Fisher Chemical/A955-4), LC-MS grade formic acid (purchased from Thermo Fisher Scientific/A117-50), Acetone (purchased from Beijing Chemical Plant /11241203810051), ammonia (purchased from Sigma/221228-500ML-A), ProteoMiner Low abundance protein enrichment Kit (purchased from Bio-Rad/1633007), trifluoroacetic acid (TFA, Purchased from Sigma/T6508-100ML).

1.2 Main Instruments

Circulating water multi-purpose vacuum pump (SHB-Ⅲ, Zhengzhou Great Wall Technology & Trade Co., LTD.); Rotary evaporation instrument (RE-52A, Shanghai Yarong Biochemical Instrument Factory); Electronic Balance (JY203, Shanghai Puchun Measuring Instrument Co., LTD.); Automated upright research microscope (BX53, Olympus (China) Co., LTD.). EASY-nLCTM 1200NA upgraded UHPLC (purchased from Thermo Fisher/LC140), QExactiveTM series Mass spectrometer (purchased from Thermo), Cryocentrifuge (purchased from Scilogex/D3024R), Freeze-dryer (purchased from Labogene/ Scan Speed 40), electrophoresis apparatus (purchased from Bio-Rad), electrophoresis tank (purchased from Bio-Rad), electronic balance (purchased from Sartorius/BSA124S), Vortex Mixer (purchased from Photosynthesis /HY-6B), Enzyme label apparatus (purchased from thermo/Multiskan FC), ice maker (purchased from Xueke), tissue grinder (purchased from Shanghai Jingxin /24 hole), ultrasonic cell crusher (purchased from Ningbo Xinzhi /JY92-11N).

1.3 Histological staining

Six mice in each group were first overanesthetized with 2.5% pentobarbital sodium and then fixed with 4% paraformaldehyde in the left lung. 24 hours after fixation, trim the tissue with a sharp blade and adjust the lung length to 0.5 cm. The tissue was then flushed overnight, followed by an alcohol gradient dehydration method: 70% ethanol for 30 minutes; 80% ethanol for 15 minutes; 90% ethanol for 15 minutes; 95% ethanol for 15 minutes; 100% ethanol for 15 minutes, twice. After xylene permeation and paraffin embedding, 4-micron paraffin sections were prepared, and then baked in an oven at 60℃ for HE staining and Mission staining. Specific HE staining steps are as follows: xylene 20 minutes, twice; 100% ethanol 5 minutes, twice; 95% ethanol for 5 minutes; 90% ethanol for 5 minutes; 80% ethanol for 5 minutes; 75% ethanol for 5 minutes; 70% ethanol for 5 minutes; Wash with distilled water for 5 minutes; Hematoxylin staining for 5 minutes; Rinse with tap water; Hydrochloric acid ethanol differentiation 15 seconds (under the number of extrusions); Rinse with running water for 15 minutes; Eosin staining for 2 minutes; Conventional dehydration; Xylene transparent, neutral gum seal. Finally, the images are collected and analyzed by microscopy.

1.4 Massion dyeing

First, xylene was treated twice for 20 minutes each time. Then it was treated with 100% ethanol twice for 5 minutes each time. Then 95%, 90%, 80%, 75% and 70% ethanol were used for 5 minutes each time. The tissues were washed with distilled water for 5 minutes and stained with hematoxylin for 5 minutes. For hematoxylin-stained nuclei, Weigert's iron hematoxylin in the Masson staining kit should be treated for 5 minutes, followed by running water. Hydrochloric acid alcohol differentiation treatment time is very short, only a few seconds, and then rinse with tap water and water for a few minutes to make it return to blue. Treat with ponceau acid fuchsin solution for 5-10 minutes, then rinse quickly with distilled water; Then it is treated with an aqueous solution of phosphomolybdate for about 3-5 minutes. Then aniline blue solution was used for re-dyeing, and the treatment was 5 minutes. Then it was treated with 1% glacial acetic acid for 1 minute; Then, they were treated with 95% ethanol twice for 5 minutes each time and anhydrous ethanol for 5 minutes each time. Finally, it was treated with xylene twice for 20 minutes. After the treatment, it is dehydrated and permeated, and the slices are removed from the xylene to dry slightly and then sealed with neutral gum. Finally, microscopically examined sections were collected and analyzed. In addition, mouse lungs were analyzed using ImageJ software and the area of lung collagen deposition was calculated to assess the degree of pulmonary fibrosis.

1.5 Extraction of interstitial lung streams

First, quantitative perfusion of the right ventricle with precooling 1*PBS until the lungs of mice turned white. The lung was then deleted, placed gently in a dish filled with precooled PBS, and gently washed to remove excess blood vessels, fat, and other tissue from the lung surface. After that, the lung tissue was carefully cut into small pieces of 1-3mm³ and placed into a centrifuge tube filled with 1ml 1*PBS, and the tubes were moved to a cell incubator at 37℃ with 5%CO2 for 2 hours. During this period, after the extracellular fluid was evenly distributed, it was absorbed, and gradient centrifugation was performed: 1000g for 5 minutes, the supernatant was taken; 4000g 10 minutes to take supernatant; 10000g for 30 minutes, take supernatant. During centrifugation, the temperature should be maintained at 4 ° C. Finally, the interstitial lung streams can be obtained by filtering through a 200-mesh filter membrane, and the remaining samples can be stored in a -80℃ refrigerator for subsequent analysis.

1.6 Proteomics and statistical analysis of lung interstitial streams

The samples were removed from the refrigerator at -80℃, transferred to a 1.5ml centrifuge tube, and added with the appropriate amount of DB protein solution (8 M urea, 100 mM TEAB, pH=8.5), and mixed by shock. Then centrifuge at 4℃ and 12000 g for 15 min, add ten mM DTT with supernatant and react at 56℃ for 1 hour, then add enough IAM and react at room temperature for 1 hour away from light. After sample treatment and extraction of enzymolysis polypeptides, 2/3 of the polypeptides were purified by a reversed-phase chromatography column and finally formed 32 fractions. The collected fractions were analyzed by the Bruker times of the Pro 2 high-resolution mass spectrometer after incorporating iRT standards (used to correct chromatographic retention time). Some of the samples were first collected based on the DDA model for establishing a spectra library, and the remaining samples were then collected through DIA mode. When selecting a window in DIA-PASEF mode, the m/z window is considered, and the collision cross-sectional window is added to improve the efficiency of ion selection. After data collection, Spectronaut software was used for DDA database search and DIA analysis, and the results were obtained and analyzed statistically.

1.7 Verify the mechanism of pirfenidone in the treatment of pulmonary fibrosis in mice

Five-week-old male BABL/C6N18 were randomly divided into three groups: normal group, model group and drug administration group. The mouse pulmonary fibrosis model was established by endotracheal injection of bleomycin (5mg/Kg body weight). The model group was orally given 0.1ml of pirfenidone suspension (200ug/ml) on the 10th day of modelling. At the same time, the normal and model groups were given PBS. On the 21st day of modelling, the left lung was fixed and stained with HE, Massion and Sirius red. The suitable lung liquid nitrogen was frozen and stored in the refrigerator at -80℃, and WB detection was performed later. The experimental results showed that compared with the normal group, both the model and drug administration groups showed different degrees of tissue inflammation and fibrosis. Compared with the model group, the inflammatory changes and fibrosis degree of lung tissue in the administration group were reduced. Compared with the normal group, the expressions of CHOP, TGF-β1, smad3, ANXA11, αSMA, and apoptosis-related Caspase 3 proteins in the model group were up-regulated. Biological experiments showed that compared with the normal group, CHOP, TGF-β1, smad3, ANXA11, α-SMA, Caspase 3 protein expression was up-regulated, and HSP-60 protein expression was down-regulated in the model group. Compared with the model group, CHOP, TGF-β1, smad3, ANXA11, α-SMA, and Caspase 3 protein expression was down-regulated, and HSP-60 protein expression was up-regulated in the administration group.

1.8 Differential protein verification

The samples and marker were placed on ice, mixed by vortex shaking after dissolution, and centrifuged for 10 s at 4,000 rpm. The glass plate was fixed in the electrophoresis tank and the electrophoresis liquid was added to the electrophoresis tank to the specified height. The sample size was calculated based on the sample protein concentration, and the corresponding sample and marker were added to the sampling tank according to the experimental design for electrophoresis. The electrophoresis process was terminated when the electrophoresis reached about 1 cm from the bottom of the bromophenol blue. The membrane was transferred using the transfer liquid and then layered into a “sandwich” structure in the following order: filter paper, PVDF film, glue, and filter paper. The sandwich structure was placed on the semi-dry film transfer instrument, and the film was transferred at 25 V for 20 min. After the membrane transfer, the PVDF membrane was removed and washed with 1*TBST at 65 rpm for 10 min three times. Finally, the primary and secondary antibodies were incubated with the closed solution. The protein bands were observed using western blot imager and ECL hypersensitive color-developing solution after washing. The protein expression levels were analyzed according to the grey values of the bands.

1.9 Total Protein Extraction and Trypsin treatment

Some sample was taken into an ultrafiltration tube and centrifuged at 14000 g for 15 min. After discarding the permeate, the ultra-filtered sample was put into a 1.5ml centrifuge tube and balanced with DB buffer (8 M Urea, 100 mM TEAB, pH 8.5). The protein solution was reduced with 10 mM DTT for 1 h at 56℃, and subsequently alkylated with sufficient IAM for 1 h at room temperature in the dark. Each protein sample was taken and the volume was made up to 100 μL with DB lysis buffer (8 M Urea, 100 mM TEAB, pH 8.5), trypsin and 100 mM TEAB buffer were added, sample was mixed and digested at 37 °C for 4 h. Then trypsin and CaCl2 were added digested overnight. Formic acid was mixed with digested sample, adjusted pH under 3, and centrifuged at 12000 g for 5 min at room temperature. The supernatant was slowly loaded to the C18 desalting column, washed with washing buffer (0.1% formic acid, 3% acetonitrile) 3 times, then added elution buffer (0.1% formic acid, 70% acetonitrile). The eluents of each sample were collected and lyophilized.

1.10 The functional analysis of protein and DEP

Gene Ontology (GO) and InterPro (IPR) functional analysis were conducted using the interproscan program against the non-redundant protein database (including Pfam, PRINTS, ProDom, SMART, ProSite, PANTHER), and the databases of COG (Clusters of Orthologous Groups) and KEGG (Kyoto Encyclopedia of Genes and Genomes) were used to analyze the protein family and pathway. DPEs were used for Volcanic map analysis, cluster heat map analysis and enrichment analysis of GO, IPR and KEGG. The probable protein-protein interactions were predicted using the STRING-db server.


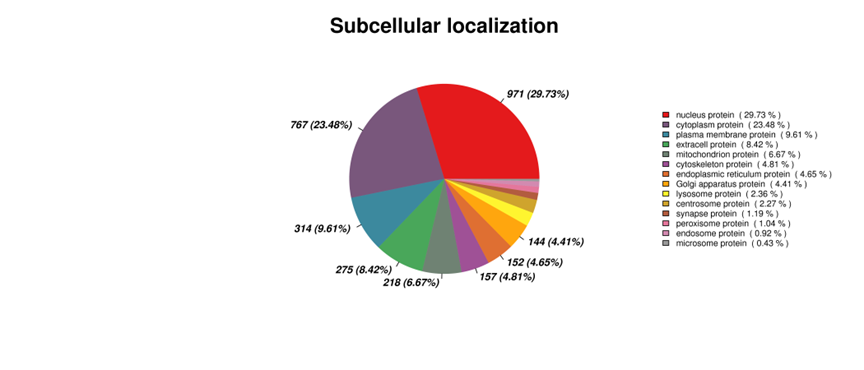


**Figure S1.** Subcellular localization of differential proteins in five groups


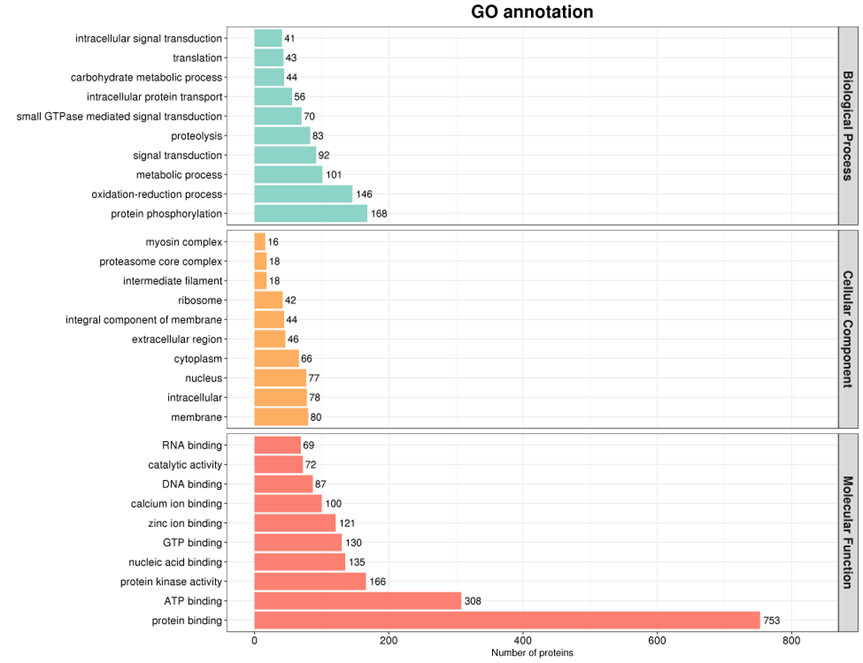


**Figure S2.** GO Enrichment Analysis of Five Groups of Total Proteins


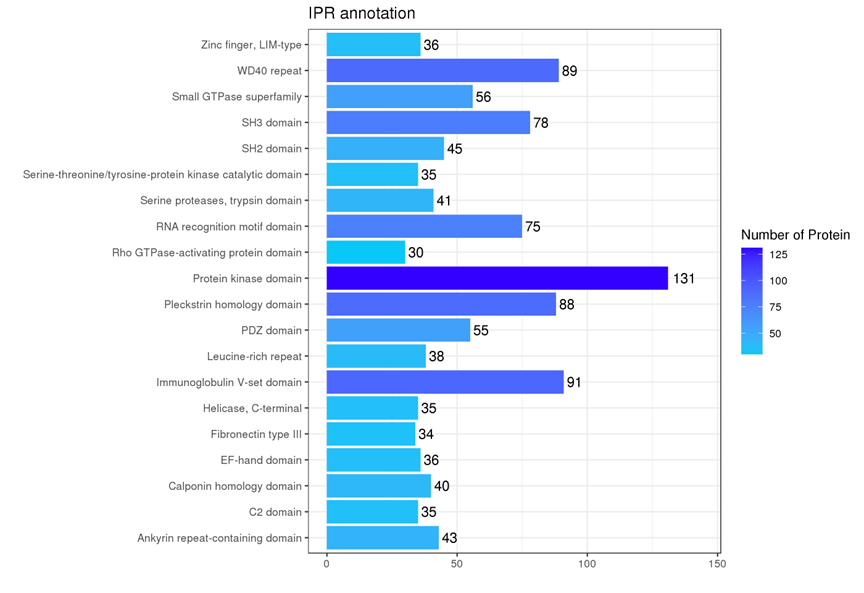


**Figure S3.** Enrichment analysis of total protein IPR in five groups


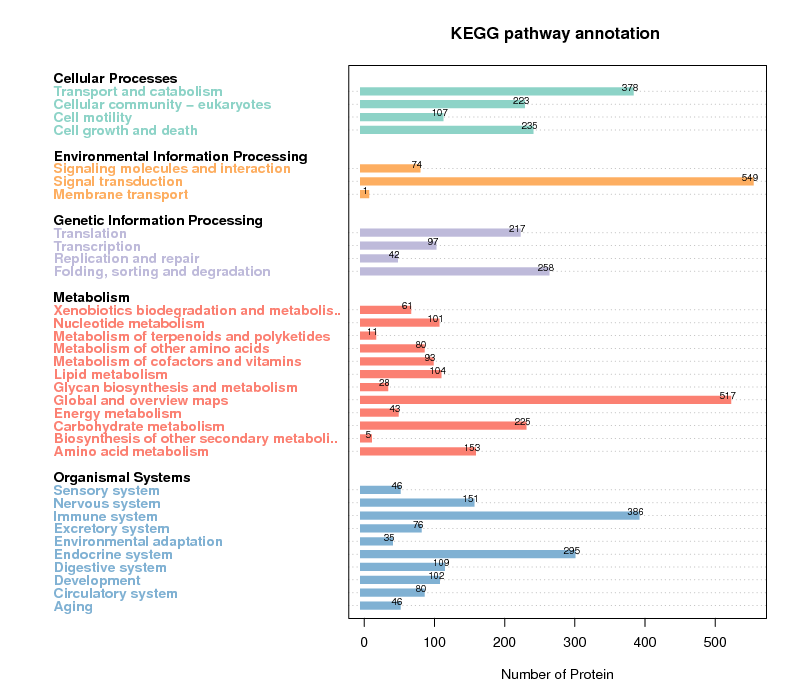


**Figure S4.** KEGG enrichment analysis of five groups of total proteins


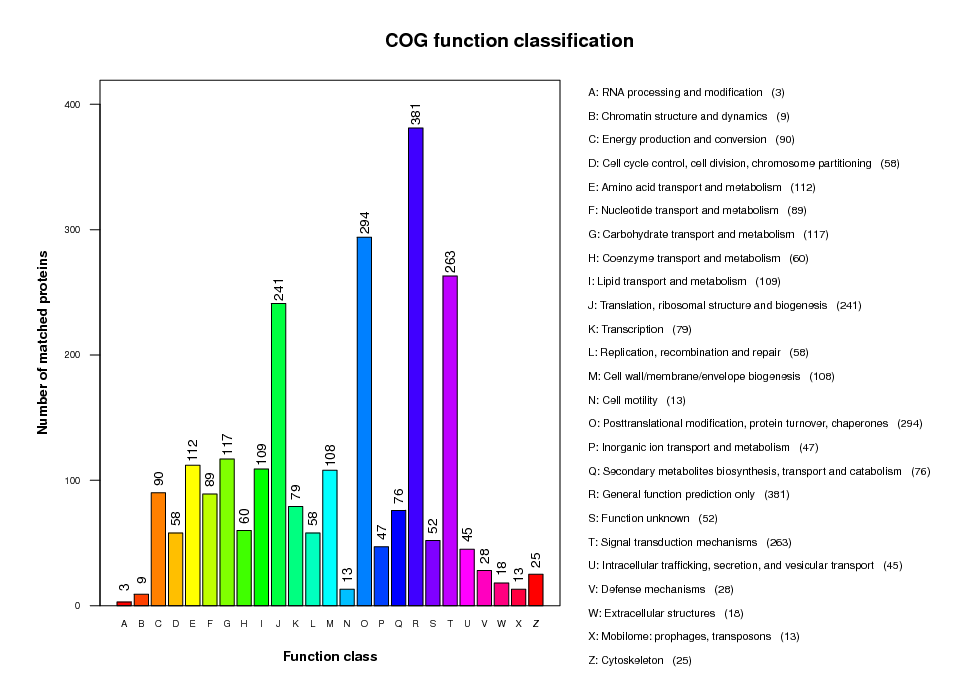


**Figure S5.** COG enrichment analysis of five groups of total proteins


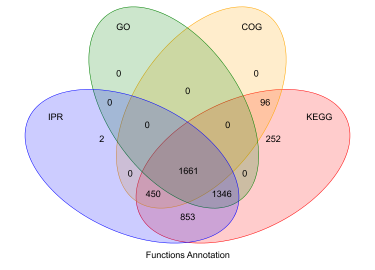


**Figure S6.** Venn diagram of protein enrichment in five groups


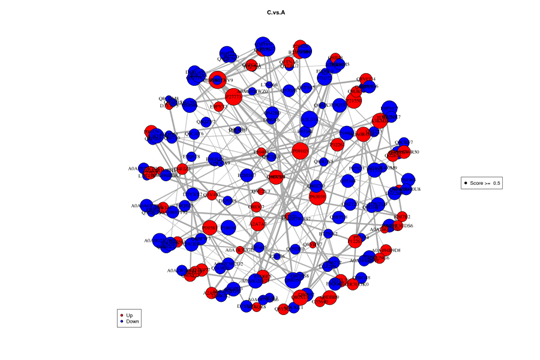


**Figure S7.** Differential protein protein interaction between the normal group and the model group after 14 days of modeling


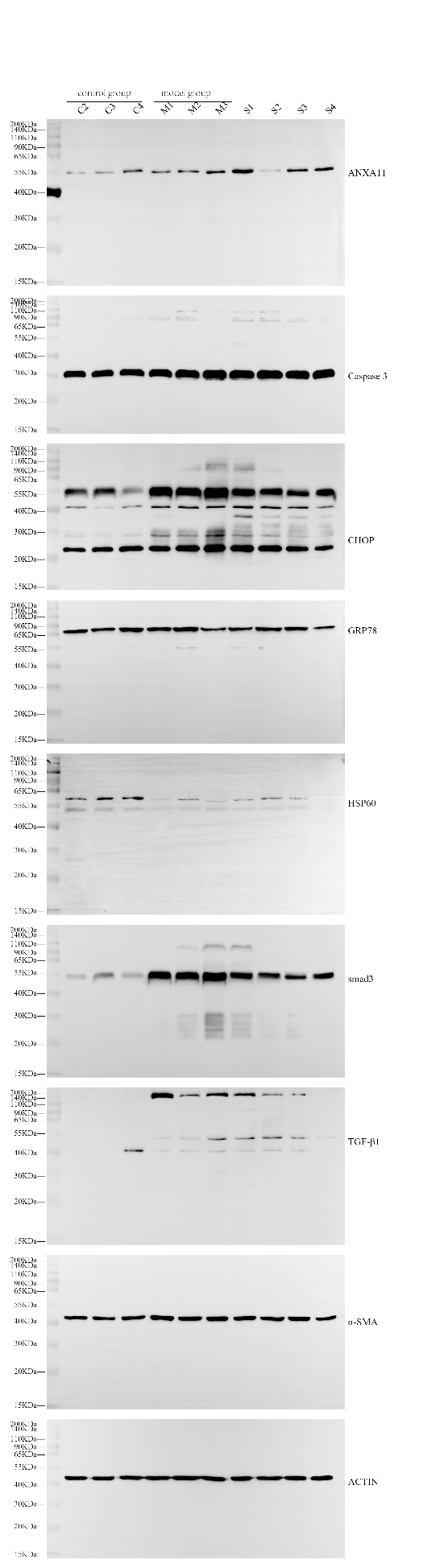

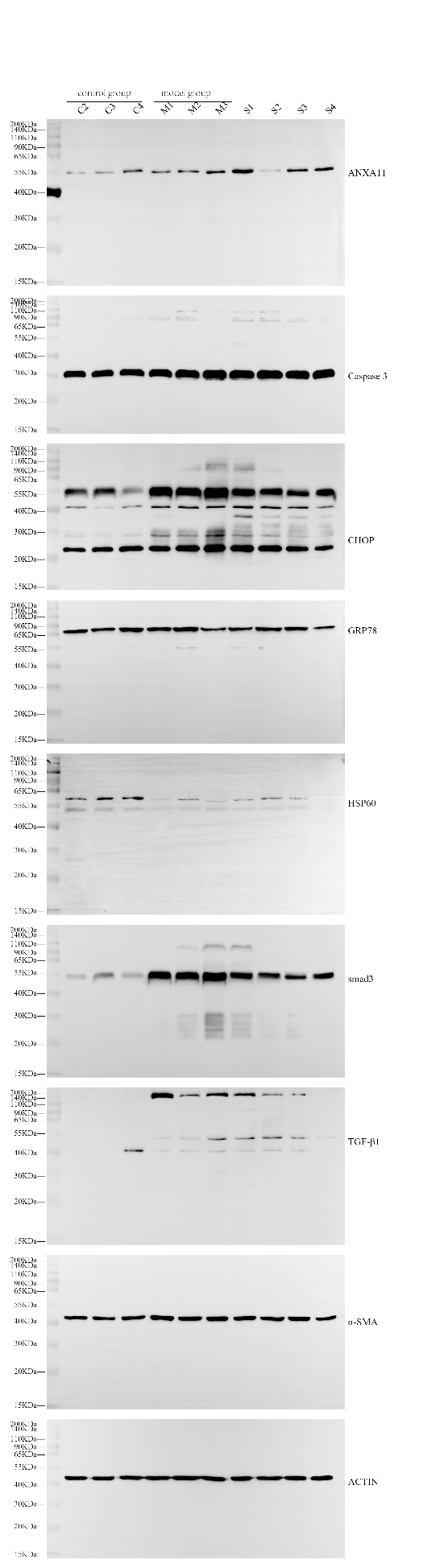


**Figure S8.** original blots

[1] Wiśniewski, Jacek R, Zoμgman. et al. Universal sample preparation method for proteome analysis. *Nature Methods* **2009**.

[2] Satpathy S, Mannan R, Ana R I. Proteogenomic Characterization Reveals Therapeutic Vulnerabilities in Lung Adenocarcinoma. *Cell* **2020**, 182(1).

[3] Zhang H, Liu T, Zhang Z. et al. Integrated Proteogenomic Characterization of Human High-Grade Serous Ovarian Cancer. *Cell* **2016**.

[4] Huang D W, Sherman B T, Lempicki R A. Bioinformatics enrichment tools: paths toward the comprehensive functional analysis of large gene lists. *Nucleic Acids Research* **2009**, 37(1):1-13.

[5] Franceschini A, Szklarczyk D, Frankild S. et al. STRING V9.1: Protein-Protein Interaction

Networks, with Increased Coverage and Integration. *Nucleic Acids Research* **2012**, 41.
